# Selection of optimal proxy locations for temperature field reconstructions using evolutionary algorithms

**DOI:** 10.1038/s41598-020-64459-6

**Published:** 2020-05-13

**Authors:** Fernando Jaume-Santero, David Barriopedro, Ricardo García-Herrera, Natalia Calvo, Sancho Salcedo-Sanz

**Affiliations:** 1 Department of Earth Physics and Astrophysics, Universidad Complutense de Madrid, Madrid, Spain; 2 Geosciences Institute (IGEO), (CSIC/UCM), Madrid, Spain; 3 Department of Signal Processing and Communications, Universidad de Alcalá, Madrid, Spain

**Keywords:** Climate change, Palaeoclimate, Computational science

## Abstract

In the Era of exponential data generation, increasing the number of paleoclimate records to improve climate field reconstructions might not always be the best strategy. By using pseudo-proxies from different model ensembles, we show how biologically-inspired artificial intelligence can be coupled with different reconstruction methods to minimize the spatial bias induced by the non-homogeneous distribution of available proxies. The results indicate that small subsets of records situated over representative locations can outperform the reconstruction skill of the full proxy network, even in more realistic pseudo-proxy experiments and observational datasets. These locations highlight the importance of high-latitude regions and major teleconnection areas to reconstruct annual global temperature fields and their responses to external forcings and internal variability. However, low frequency temperature variations such as the transition between the Medieval Climate Anomaly and the Little Ice Age are better resolved by records situated at lower latitudes. According to our idealized experiments a careful selection of proxy locations should be performed depending on the targeted time scale of the reconstructed field.

## Introduction

Decades of scientific fieldwork have left abundant proxy datasets to reconstruct the climate of the past. They provide information about climate changes at local and regional scales, and have been pooled to derive Climate Field Reconstructions (CFRs) of the last millennium^1,2^. CFRs are spatially-resolved reconstructions of climate variables generated from different paleoclimate archives. These reconstructions are affected by several sources of uncertainty^3,4^ related to the reconstruction method (e.g. dimensionality, dependence on parameters, frequency coherence)^3^, the underlying proxy observations^5^ (e.g. observational error, irregular chronologies, observed resolution), their links with the target field (e.g. multivariate signals, stationarity, spatial and temporal covariance, resolved resolution and seasonality), or the non-uniform spatial distribution of the observing network^6^. While most of these uncertainties have been thoroughly studied, spatial biases induced by sparse sampling locations are still not well understood. Due to the limited availability of paleoclimate archives, most proxy records are restricted to land, mainly the middle latitudes of the Northern Hemisphere, while there are extensive un-sampled regions in the Southern Hemisphere and high latitudes. This unbalanced distribution of paleoclimate records induces a spatial bias in global CFRs that remains poorly quantified.

Circumventing this issue requires, in first place, a wise selection of proxies^7^. Due to the increasing availability of high-resolution records, the initiative Past Global Changes (PAGES-2k) has scrutinized thousands of temperature-sensitive proxies, releasing a global archive with paleoclimate records for the last two millennia^8^. This network has been recently exploited to derive global multi-proxy CFRs of annual temperature from a suite of reconstruction methods, including model-based assimilation schemes similar to those employed in modern reanalyses^9–11^. However, as these proxies are unevenly distributed, the spatial bias is still present. Selecting records strategically situated over key regions that capture the diversity of global patterns and simultaneously minimize the spatial bias of the observing network represents a significant challenge^12,13^. Problems of this kind cannot be directly solved by testing all possible combinations due to their high dimensionality. Efforts to address this issue have only been attempted with sequential approaches (i.e. step-wise solutions based on local search procedures that perform incrementally by adding the proxy records with the best performance). Differently, a branch of artificial intelligence based on soft-computing techniques has recently emerged to solve high dimensional problems^14,15^. For instance, biologically-inspired methods such as evolutionary algorithms^16^ are global search procedures that outperform sequential approaches in the task of finding optimal solutions to the representative selection problem within large and complex datasets^17,18^.

In this study we deal with the spatial bias in global CFRs of annual temperature arising solely from the non-homogeneous distribution of the currently available network of proxy records for the last millennium. To better constrain this bias, other sources of uncertainty are avoided by using the Community Earth System Model Last Millennium Ensemble^19^ (CESM-LME) as a surrogated reality, where pseudo-proxies (synthetic temperature series from the target simulation) matching the locations of the PAGES-2k archive are artificially generated (see Methods). For these idealized conditions of the PAGES-2k proxy network (complete observational availability over time, univariate signals without observational error, stationary relationships, etc.) we generate global CFRs of annual temperature for the last millennium simulation of the CESM-LME that are biased by the uneven distribution of real proxies. CFR techniques^20^ are then coupled with an evolutionary algorithm to explore if optimized subsets of PAGES-2k locations can be used instead without sacrificing the reconstruction skill. These pseudo-proxy experiments allow us to address the following questions in the perfectly known model’s world: Can we quantify the spatial bias due to the uneven distribution of records? Do we need all available records of the PAGES-2k network to maximize the skill of global temperature field reconstructions of the last millennium? If not, how many records are required to reconstruct the temperature of the last millennium without degrading the skill? Can we find a subset of PAGES-2k proxy locations that reduces the spatial bias of the full-proxy network?

## Selection of representative locations

Annual temperature global patterns of the first full-forcing CESM-LME member simulation spanning the 850–2005 period of the Common Era (CE) are chosen as the target fields to reconstruct from pseudo-proxies^21^ at the 569 grid-points matching the locations of the PAGES-2k archive. The accuracy of the CFR is quantified as its Root-Mean-Square Error (RMSE) against the spatially-resolved global temperature patterns of the target simulation. Most experiments have been set under idealized conditions by using perfect pseudo-proxies directly assembled from the simulated temperature series of the target simulation. Unlike real reconstructions, our CFRs are only affected by biases due to the spatial distribution of paleoclimate archives and the CFR methodology. This approach avoids other sources of uncertainty, allowing us to better constrain spatial biases, which can be inferred from changes in the reconstruction skill arising from different rearrangements of the pseudo-proxy network.

An evolutionary algorithm is herein coupled with a CFR method to find subsets of N < 569 proxy locations of the PAGES-2k archive that minimize the RMSE of annual temperature field reconstructions. We use the Coral Reef Optimization with Substrate Layers^22^ (CRO), an ensemble evolutionary algorithm based on the reproduction of corals in a reef, which combines search operators within a set of different solutions (Methods). It has already been used to find sets of locations that best describe spatially resolved climate fields such as wind speed^23^ or temperature^17^, and their results have been applied to improve solar and wind power forecasts^23,24^ at local scales. Different to sequential approaches, the CRO searches for N solutions simultaneously, starting from an initial seed that evolves in subsequent iterations depending on its performance in terms of a health function (minimization of the RMSE).

To test the sensitivity of the results to the CFR method, two different CFR techniques have been employed (Methods): The Analogue Method^20^ (AM) and the Canonical Correlation Analysis^25^ (CCA). The 1850–2005 period of the first member of CESM-LME is used for the calibration of the CCA (similar results are obtained for shorter calibration periods, e.g. 1900–2005), while the remaining members of the same model ensemble are employed as a pool of analogues to derive the CFR with AM. Our results for the first member have also been tested in other realizations of the same model, a different model, and more realistic datasets, as explained below. To add complexity and more realistic conditions, experiments with additional sources of uncertainty were also performed. They allow us to test the robustness of the results and benchmark the magnitude of the spatial bias against that arising from other sources of uncertainty (the effect of multiple uncertainty sources should not be considered additive, though). In particular, we account for the amplitude of the observational error (Signal to Noise Ratio, SNR) by adding different levels of red noise to the perfect pseudo-proxies with a lag-1 autoregressive model (Methods). Acronyms of techniques and experiments have been compiled in Table S1.

**Figure 1:**
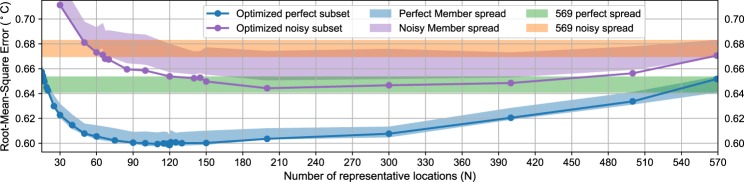
RMSE of the 850–2005 CE global temperature fields reconstructed with CRO-AM as a function of the number of selected perfect (blue) and noisy pseudo-proxies (purple) of the PAGES-2k network. The green and orange shades represent the 13-member reconstruction skill spread obtained with all (569) perfect and noisy pseudo-proxies (with SNR of 1) of the PAGES 2-k network. The blue-shaded area represents the spread obtained by using the optimized subset of N pseudo-proxies obtained for the first CESM-LME member to reconstruct the remaining members of the ensemble. The purple-shaded area is the same as the blue-shaded one but for reconstructions using noisy pseudo-proxies with SNR of 1. All shades depict 2 standard deviations with respect to the mean.

The AM-reconstructed global temperature fields using all perfect pseudo-proxies leads to a measurable RMSE of 0.65 °C, which should be ascribed to uncertainties in both the limited number and distribution of records (spatial bias) and the CFR method. For comparison, the RMSE derived from noisy pseudo-proxies with a SNR of 1 is only increased by 0.02 °C, whereas a SNR of 0.5 degrades the reconstruction skill by 0.07 °C (Table 1). To quantify the spatial bias, we derived new CFRs from reduced subsets of N perfect pseudo-proxies constrained by CRO-AM. For all optimized solutions of N records tested, the CRO-AM generates more skillful reconstructions than selecting subsets of N perfect pseudo-proxies at random (Fig. S1). Consequently, the improvement obtained with CRO-AM is not related to the reduction of random error, but a meaningful identification of representative locations for the reconstruction of temperature fields. A minimum optimized set of 17 perfect pseudo-proxies (CRO-MIN) is enough to obtain global temperature fields with the same RMSE as the full-proxy reconstruction (Fig. 1). Therefore, under ideally perfect conditions, skillful reconstructions can be achieved even when the number of records is reduced up to one order of magnitude with respect to the PAGES-2k network. But, can we reduce the spatial bias of the full-proxy reconstruction with a subset of well-distributed records larger than CRO-MIN? Our idealized experiments indicate that CFRs from an optimal selection of records can outperform the skill of the full-proxy reconstruction (Fig. 1). In particular, the RMSE of full-proxy temperature field reconstructions can be reduced up to 0.05 °C after selecting an optimal set of 120 perfect pseudo-proxies (CRO-OPT). Note that the associated error (0.60 °C) approaches that obtained from a full global grid coverage of perfect pseudo-proxies (0.54 °C). Besides minimizing the RMSE of the CFR for the first member, CRO-OPT locations also yield comparable levels of performance for the other members of the ensemble (Fig. 1, shading). This indicates that the selected network is representative across the ensemble, although the comparatively higher RMSE in those members points to additional improvements if the CRO-AM was applied separately to each realization. Similar estimates of the spatial bias are also found for CRO-CCA reconstructions (a RMSE reduction of ~0.05 °C for an optimized subset of 120 perfect pseudo-proxies), stressing the robustness of the results with respect to the CFR technique. Indeed, the RMSE variation with the size of the selected network shown in Fig. 1 is also reproduced by CCA reconstructions based on the locations selected by the CRO-AM (Fig. S2).

**Table 1:** RMSE of global temperature fields for 850–2005 CE (in °C) using CRO-AM reconstructions with N representative AR(1) pseudo-proxies of the PAGES-2k network and different SNR.

		SNR		
		∞	1	0.5
N	30	0.62	0.71	0.82
	200	0.60	0.64	0.71
	569	0.65	0.67	0.72

RMSE are calculated between the spatially-resolved global temperature fields of the corresponding reconstruction and the target simulation (first member of the CESM-LME) for 850–2005 CE.

The reduction of the RMSE with CRO-OPT is not negligible, since it is equivalent to doubling the SNR in the full-proxy reconstruction (Table 1). In the case of noisy pseudo-proxies with SNR of 1, the minimum number of records necessary to reach the same skill as their full-proxy reconstruction increases with respect to that obtained from perfect pseudo-proxies (60 instead of 17 in CRO-MIN). Still, it is possible to find representative subsets of 150 noisy pseudo-proxies that outperform the skill of the complete network of noisy pseudo-proxies (Fig. 1, lines). The same conclusion is obtained for pseudo-proxies with more realistic components of random noise (SNR = 0.5), although previous experiments have shown that this level of SNR provides pseudo-proxy CFRs with lower skill than real-world reconstructions^5^. In addition, the noisy subsets that optimize the CFR of the first member are able to reduce the reconstruction error obtained for the other members of the ensemble from their complete networks of noisy pseudo-proxies (Fig. 1, purple shading). For certain conditions (SNR = 1 in Fig. 1), optimized subsets of noisy pseudo-proxies can even outperform the skill of the full network of perfect pseudo-proxies. Therefore, with all other sources of uncertainty being absent, spatial biases induced by a non-uniform distribution of high-quality (ideally perfect) proxies can potentially be larger than those obtained from a reduced subset of well-distributed noisy proxies with SNR of 1. Additional exploratory tests for other sources of uncertainty show that the reconstruction skill of the CRO-OPT subset does not fall below that of the full-proxy network, even if some assumptions of our idealized experiments are intentionally violated (e.g. by imposing the observed temporal availability of PAGES-2k records).

**Figure 2:**
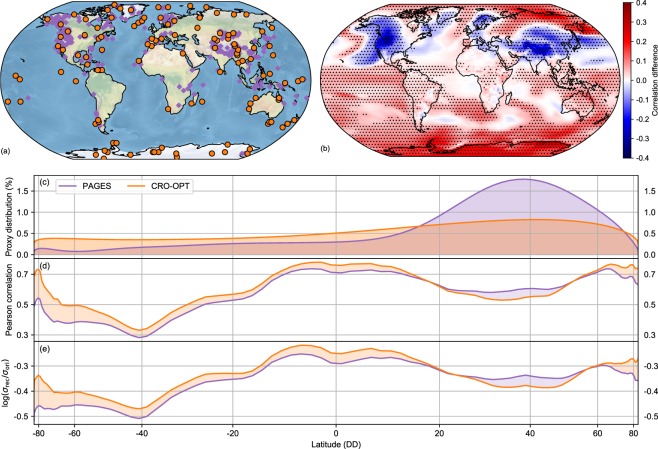
Performance of the CRO-AM reconstruction with the optimal subset of PAGES-2k records (CRO-OPT). (**a**) Spatial distribution of CRO-OPT records (orange dots) obtained from the full PAGES-2k network (purple diamonds). (**b)** Spatial correlation difference between the temperature reconstructions with CRO-OPT and all perfect pseudo-proxies. Stippling points illustrate significant correlation differences (p < 0.05). Kernel density estimation of the (**c)**, Normalized latitudinal distribution of records (in % with respect to the total number of pseudo-proxies) for the CRO-OPT subset (orange) and the full-proxy PAGES-2k network (purple). (**d)** Latitudinal mean Pearson correlations for the CRO-OPT (orange) and full-proxy (purple) reconstructions. (**e)** Latitudinal logarithm of the standard deviation ratio for the CRO-OPT (orange) and full-proxy (purple) reconstructions (σ_rec_) compared with the target simulation (σ_ori_). The latitudinal axis is proportional to the effective area.

Most of the selected locations in CRO-OPT are situated at high latitudes (Fig. 2a), stressing the importance of Arctic^26,27^ and Antarctic^28^ regions. Although there is not a unique solution, the distribution of optimal locations does not vary substantially with the number of selected records (cf. Figs. 1a and S3), and the CFR method (Fig. S4), even if we use noisy pseudo-proxies (Fig. S5) or area-weighted fields (Fig. S6). The improvement of the CRO-OPT reconstruction is also reflected at local scales (Fig. 2b), implying that small changes in the global skill (Table 1) hide large regional improvements. The Pearson correlation coefficient of the target simulation with this reconstruction is significantly higher than with the full-proxy reconstruction for almost the entire Southern Hemisphere and the Arctic, and only performs worse in regions where the complete network presents high density of perfect pseudo-proxies. This is consistent with the spatial pattern of the RMSE difference between the CRO-OPT and full-proxy reconstructions (Fig. S7). Interestingly, highly sampled regions by the PAGES-2k network tend to coincide with areas of low spatial autocorrelation (Fig. S8). As such, the regional details of the temperature field over these regions are better captured by the denser full-proxy network than by CRO-OPT. However, our experiments indicate that this regional improvement of the full-proxy network is attained with sacrifices in its global performance. These results suggest that spatial clusters of proxy records may debase the skill of spatially-resolved global temperature reconstructions. Note that the CRO-OPT distribution (Fig. 2c) is not simply a uniform one, and fewer points are often selected over areas with high spatial autocorrelation (e.g. the tropics). Still, its reconstruction leads to generalized latitudinal improvements in terms of correlation (Fig. 2d) and variability (Fig. 2e). These strategic locations also retrieve skillful reconstructions of area-weighted global mean temperatures (GMT) for the last millennium. The GMT of the reconstruction generated with CRO-OPT shows high skill (RMSE of 0.09 and R^2^ of 0.88), improving that from the full-proxy reconstruction (RMSE of 0.16 and R^2^ of 0.71).

Recall that the selected locations represent an optimal subset of the PAGES-2k network for the specific conditions and target field of our idealized experiments. To test the independence of these results from the model employed, the CRO-AM experiment has also been run in the CCC400 model ensemble^10,29^ (Methods). The results for an optimized subset of 120 locations (the same number as in CRO-OPT) are depicted in Fig. S9. The spatial distribution of selected locations, the improvement of the correlation at high latitudes and part of the Pacific Ocean, and the latitudinal correlation and variability patterns resemble their CESM-LME counterparts presented in Fig. 2. A similar distribution persists in out-of-sample validation tests where the CCC400 ensemble is used as a pool of analogues to reconstruct the first member of the CESM-LME (Fig. S10), indicating that the selection of representative locations is not sensitive to the training data used in the process.

**Figure 3:**
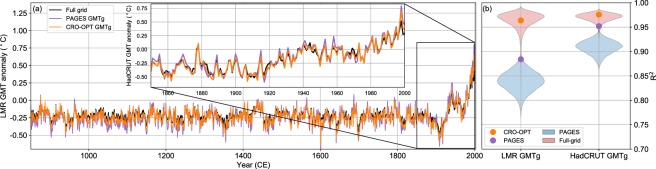
Estimates of GMT anomalies (°C) for the last millennium as inferred from selected subsets of the PAGES-2k network. (**a**) GMT anomalies from the LMR for 850–2000 CE (Inset a, GMT anomalies from HadCRUT4 for 1850–2000 CE). Purple and orange lines show the GMTg of these datasets, defined as the area-weighted temperature mean for the grid points matching the PAGES-2k and CRO-OPT locations, respectively. All anomalies are computed with respect to the 1961–1990 baseline. (**b)** Coefficient of determination between the time series of GMT and GMTg from PAGES-2k (purple) and CRO-OPT (orange) locations. Violins illustrate the distributions obtained for 10000 subsets (with the same size as CRO-OPT) of randomly-selected locations from the PAGES-2k network (blue) and the full global grid (red).

We have also performed more stringent tests to assess whether the CRO-OPT locations inferred from the CESM-LME are also informative of GMTs in more realistic datasets. The exercise has been applied to instrumental temperature data based on HadCRUT 4.2^30^ for the post-industrial period (1850–2008 CE), as well as to proxy-based temperature reconstructions for the last millennium (850–2000 CE) provided by the Last Millennium Reanalysis (LMR, Methods). These products are independent from the CCC400 and CESM-LME, except for the fact that the LMR uses prior state estimates from an earlier version of the CESM atmospheric model. For each of these datasets, we computed first guess GMTs (GMTg hereafter) as the area-weighted mean temperature for two different sets of locations: the CRO-OPT, previously obtained with the CESM-LME, and the complete PAGES-2k network. Note that GMTg are directly obtained from the temperature series at these specific locations, without reconstructing the global temperature fields of the respective dataset. Although this approach is not ideal, and differs from global aggregation strategies typically employed for the computation of GMTs (area-weighted global means from re-gridded fields), it gains importance in the light of incomplete coverage and time-varying availability of global records. Figure 3 shows how the HadCRUT 4.2 and LMR GMTs are overall consistent with their GMTg. In both cases, the coefficients of determination (R^2^) are higher for CRO-OPT than for the complete PAGES-2k network, and they are also significantly higher than selecting random sets of locations from the PAGES-2k archive (Fig. 3b). This evidences the representativeness of CRO-OPT in observations and real-world reconstructions.

### Reconstruction of internal and externally-forced temperature patterns

**Figure 4:**
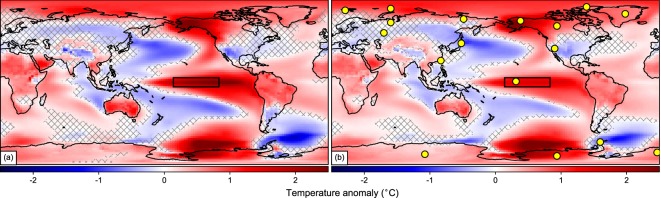
Reconstruction skill of internal variability patterns with the CRO-MIN subset of PAGES-2k records. Composite of annual temperature anomalies (°C, with respect to 850–2005 CE) for El Niño events in (**a)**, the target field (the first CESM-LME full-forcing member). (**b**) The reconstructed field from the CRO-MIN subset of perfect pseudo-proxies of the PAGES-2k network (yellow dots). For each panel, crosses depict non-significant temperature differences at 95% confidence level with respect to its corresponding climatology inferred from a bootstrap of 10000 random samples. El Niño events are defined as years of the target simulation with standardized temperatures above the 95^th^ percentile at El Niño-3.4 region (black square).

Despite its improvement, the optimized selection of locations is constrained by the original distribution of proxy records in the PAGES-2k archive, which displays limited coverage and a spatial bias towards mid-latitude land areas of the Northern Hemisphere, with very few proxies over oceanic regions. Therefore, we explored the skill of this reduced subset to capture internal variability and externally forced signals in the global temperature patterns. This also allows us to assess whether the selected locations have physical meaning or simply represent an optimized statistical distribution. El Niño-Southern Oscillation (ENSO) is chosen as an example of the former. The perfect pseudo-proxy CFR with CRO-OPT locations explains more than 80% of the SST variance over El Niño 3.4 region for the pre-industrial period of the target simulation, and even the more constrained CRO-MIN reconstruction captures global ENSO teleconnections reasonably well (Fig. 4). Taking into account that ENSO is the main mode of internal variability on interannual time-scales, it is surprising that only one of the selected locations in CRO-MIN is over the tropical Pacific (Fig. 4b). However, the spatial autocorrelation of temperature variations in this region is among the highest of the globe (Fig. S8), which arguably reduces the effective number of records required to reproduce them. The ability of this subset to capture ENSO signals can be further explained by the optimized distribution, with some of the selected locations laying in areas strongly affected by ENSO teleconnections, such as the nodes of the Pacific North/South American pattern^31^. This suggests that the CRO-AM tends to prioritize those records strategically situated over major climate teleconnections, which together explain a large fraction of the global temperature variance. Indeed, the CRO-OPT subset can also capture large-scale internal modes of atmospheric circulation variability, such as the Northern Annular Mode (NAM), herein reconstructed from the annual mean sea level pressure (SLP) fields of the best temperature analogue years (Fig. S11). The reconstructed NAM series follows the simulated variations in the target run, although with considerable uncertainty, and a tendency to underestimate the target amplitude. The latter suggests that, despite being informative, networks specifically optimized for a given target field do not necessarily represent the optimized solutions for the reconstruction of other fields.

**Figure 5:**
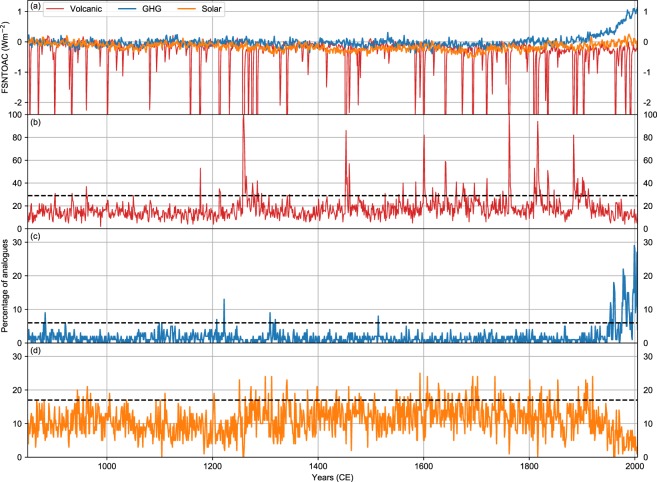
Detection of external forcings in the reconstruction with the CRO-OPT subset of the PAGES-2k network. (**a**) Annual mean clear-sky net solar flux at top of the atmosphere for three single-forcing ensemble simulations. Percentages of the 100 best analogue years selected from CRO-OPT with the same dominant forcing as in the given year of the target simulation. (**b)** volcanic, (**c)** greenhouse gases, and (**d)** solar forcing. Black dashed lines depict the significance thresholds above which there is an instantaneous detection of forced signals attributed to the given forcing (see Methods).

As for the external forcings, the constrained reconstructions from subsets of perfect pseudo-proxies also capture the timing and spatial fingerprint of the simulated cooling response to major volcanic events of the Last Millennium (see the target and reconstructed field from CRO-OPT following the Tambora^32^ eruption, Fig. S12). Interestingly, CRO-AM replicates these forced responses by choosing years from the CESM-LME with significant volcanic activity. This suggests that the CRO-OPT reconstruction is able to discern the volcanic fingerprint in the global temperature patterns from the internal variability. Therefore, in a more general context, we have assessed if CRO-OPT can detect externally forced responses and attribute them to the responsible forcing. This is done by first assigning each year of the CESM-LME to a dominant factor (i.e. the one with the largest radiative forcing in the top of the atmosphere for that year) by using single forcing simulations of the CESM-LME (Methods). Then, for each year of the target simulation we count the detected frequency of each external forcing in the 100 best analogue years selected using CRO-OPT locations and test whether this frequency is significantly larger than that expected by random chance. The results for key forcings of the last millennium (Fig. 5) confirm that CRO-OPT is able to detect some forced signals and assign them to the right forcing. This is true for years following large volcanic eruptions (e.g. Tambora 1816) and periods with high volcanic activity (e.g. the 18^th^ century). Similarly, the conspicuous warming of the second half of the 20^th^ century is preferentially reconstructed from years with strong anthropogenic forcing, so that this signal can be attributed to increasing concentration of greenhouse gases. On the contrary, solar signals on annual time scales are not well discernible from the internal variability. Note, however, that it does not mean a lack of solar forcing signals since our approach is instantaneous (based on annual forcings and temperature patterns) and hence it does not take into account lagged or low-frequency responses to external forcings.

### Insights on past anomalous periods

We now focus on longer time-scales and explore how well the CRO-AM reconstructions capture key anomalous periods of the past, such as the Medieval Climate Anomaly^33^ (MCA, 950–1250 CE) and the Little Ice Age^34^ (LIA, 1450–1850 CE). Although model simulations and proxy-based reconstructions agree on the existence of a global mean temperature difference between both periods, there are discrepancies in its magnitude and spatial pattern, which are still not well understood^35,36^. Models usually yield a variety of spatial temperature patterns for the MCA-LIA transition as well as weaker differences than proxy-based reconstructions^35^. The MCA-LIA GMT difference for our target simulation is 0.19 °C, similar to the remaining members of the CESM-LME. However, this value is halved in the reconstructions obtained with CRO-MIN, CRO-OPT and the complete PAGES-2k network, even if we use ideally perfect pseudo-proxies (Table S2). The attenuation of the global warming amplitude is further evidenced by the spatial pattern of temperature differences depicted in Fig. S13.

**Figure 6:**
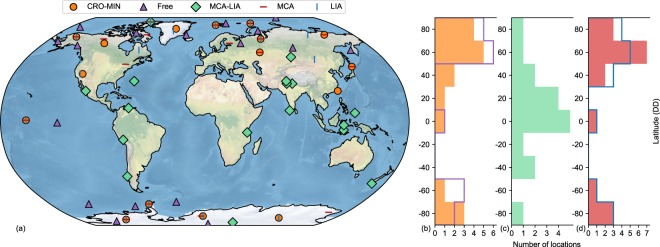
Distribution of representative locations for different experiments with perfect pseudo-proxies and the CRO-AM. (**a**) 2-D and (**b–d)**, latitudinal distribution of optimized subsets of perfect pseudo-proxies (with the same size as CRO-MIN) selected with the CRO-AM for different optimization problems. Optimized reconstruction of the global annual temperature fields of the last millennium from locations constrained to the PAGES-2k network (CRO-MIN, orange) and from an unconstrained selection (Free, purple). Optimized subsets of the PAGES-2k network for the reconstruction of the global annual temperature fields of the MCA (red) and LIA (blue) periods separately, and the spatial pattern of the mean temperature difference between the MCA and LIA (MCA-LIA, green). Latitudinal distribution of (**b)** CRO-MIN (orange shading) and Free (purple line). (**c)** MCA-LIA. (**d)** MCA (red shading) and LIA (blue line).

To deep further into the causes of the MCA-LIA underestimation, we first assess whether they stem from a limited coverage of the current PAGES-2k archive. To address this question, the CRO-AM was run to find the same number of representative locations as in CRO-MIN, but from the full grid of CESM-LME (i.e. without constraining the search to the PAGES-2k network, Free run in Fig. 6). The resulting distribution of selected locations has similarities with CRO-MIN, stressing the relevance of high latitudes (Fig. 6b). Therefore, we conclude that climatically representative regions for the last millennium are sufficiently covered by the PAGES-2k archive. This also holds for the MCA and LIA periods, whose reconstruction skills with CRO-MIN are comparable to those obtained for the entire period of the last millennium. Dedicated experiments to find specific subsets of the PAGES-2k network that optimize the CFR for the MCA and LIA separately (MCA and LIA runs in Fig. 6) also bear strong resemblance with the distribution obtained in the last millennium experiment (CRO-MIN). Indeed, good analogues of the MCA and LIA global patterns can be found through most of the last millennium, although with reduced probability during exceptionally cold and warm intervals, respectively, including the 20^th^ century (Fig. S14). Interestingly, MCA patterns do not resemble those of the 20^th^ century, indicating distinguishable MCA signatures with respect to the organized global warming of the 20^th^ century in the model world, which is consistent with previous findings^35^.

Therefore, the question is if there is any subset of PAGES-2k locations that can reproduce the MCA-LIA pattern (i.e. the spatial pattern of temperature differences on multi-centennial scales). This has been accomplished by modifying the health function of the CRO algorithm in order to find subsets of perfect pseudo-proxies that minimize the spatial RMSE of the MCA minus LIA temperature pattern (MCA-LIA run in Fig. 6). The results of this experiment reveal that it is possible to find a subset of the same size as CRO-MIN that matches the MCA-LIA pattern with almost the same amplitude as in the target simulation (0.15 °C). However, this improvement in reproducing the MCA-LIA pattern is made by sacrificing the reconstruction skill on annual scales, with the RMSE increasing from 0.65 °C in CRO-MIN to 0.80 °C in the MCA-LIA run. Furthermore, MCA-LIA locations are mainly situated over tropical and extra-tropical latitudes (Fig. 6c), leading to a completely different latitudinal distribution compared to that in the annually-resolved MCA, LIA, CRO-MIN or Free experiments.

**Figure 7:**
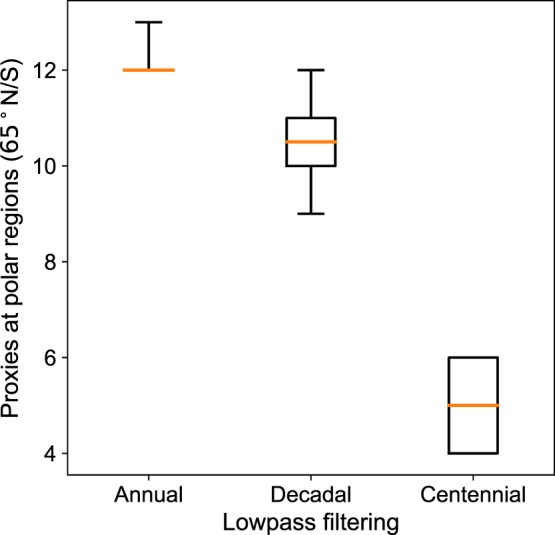
Number of records at polar regions (latitudes above 65°N/S) in optimized subsets of 20 perfect pseudo-proxies of the PAGES-2k network for CRO-CCA reconstructions of global temperature fields on different time scales. The CRO-CCA has been run to find subsets of 20 perfect pseudo-proxy locations of the PAGES-2k network that optimize the reconstruction of the global temperature fields of the first CESM-LME member at annual, decadal and centennial time scales by using a 1-, 10- and 100-year low-pass filter smoothing, respectively. The distributions include 200 different optimized solutions from 5 CRO-CCA runs (40 best solutions of each run were kept). Boxes represent the median (orange line) and interquartile ranges of the distribution, with whiskers denoting extreme solutions.

These results indicate that perfect pseudo-proxy locations that optimize the reconstruction in the high-frequency do not necessarily bring an improvement in the lower frequencies of the spectrum. To support this statement, we have performed additional experiments where annual temperature fields of the target simulation have been low-pass filtered with 10- and 100-year running windows. The CRO-CCA has been run this time to find optimized subsets of the PAGES-2k network targeting the CFR on each time scale. Figure 7 shows how the number of selected records at high latitudes (poleward of 65°) significantly decreases for lower frequencies. This agrees with the optimized distribution for the reconstruction of the MCA-LIA spatial pattern shown in Fig. 6. As the large interannual variability at high latitudes was removed for the low-pass filtered fields, we hypothesize that fewer high-latitude records are needed to reconstruct long term temperature variations. As a consequence, the optimality of the locations selected for the annual CFR is progressively lost towards the lower frequencies of the spectrum. This is further supported by the power variance spectrum of GMT reconstructions obtained with different subsets of the PAGES-2k network (Fig. S15), which shows similar performance of the CRO-OPT and full-proxy reconstructions on longer time scales for all ensemble members. Both networks underestimate the low frequency variations of the true GMT, in agreement with the reduced amplitude of their MCA-LIA reconstructions. Although the ultimate causes are unclear and require dedicated studies in more realistic conditions, our experiments point towards a significant dependence of the optimal subset of PAGES-2k locations on the time scale variations of the target field that are pursued by the CFR.

By coupling an evolutionary algorithm to different reconstruction methods and using pseudo-proxies from two model ensembles, we have quantified a measurable spatial bias in global temperature reconstructions due to the non-uniform distribution of currently available paleoclimate records. In our idealized experiments, an optimized selection of pseudo-proxies from the full network can be performed without sacrificing the skill of the reconstruction. For all experiments performed, the skill of the full-proxy reconstruction can even be improved by using subsets of pseudo-proxies strategically situated over representative locations. The set of optimal locations highlights the importance of polar regions and major teleconnections areas to reconstruct annual global temperature patterns, and captures the global responses to external forcings and internal variability. However, while annual temperature fields are well described by high-latitude records, low frequency fluctuations such as the MCA-LIA transition are better represented by pseudo-proxies situated at lower latitudes. As the optimal distribution of records varies with the spectral frequency of the target field, this advises against pooling together proxies that resolve different time scales and encourages further research for weighting records depending on their location and response resolution. Our results are robust to the reconstruction methodology and the ensemble member or model employed, and they hold for more realistic pseudo-proxy experiments and datasets. Still, important assumptions have been made and all uncertainty sources have not been considered, which calls for caution when extrapolating these results to real reconstructions. In this endeavor, further experiments are strongly encouraged in order to account for additional sources of uncertainty not included herein, such as the temporal availability of the observing network or the non-univariate dependency of proxy records to temperature variations. Considering all sources of uncertainty would require dedicated but feasible implementations in the CRO algorithm, potentially bringing changes in the structure of the optimized networks reported herein. Therefore, our promising results will hopefully make the leap to the real world, where more skillful reconstructions could be attempted through optimal subsets tailored to the temporal availability of the observing network and optimization functions tackling with the climate signal and response resolution embedded in different paleoclimate archives. Finally, our approach paves the way for determining valuable regions to carry out future measuring campaigns of proxy records^37^, which can be elucidated by mapping areas with natural paleoclimate archives that are underrepresented in current proxy networks.

## Methods

### Data description

The PAGES-2k multi-proxy database is composed of 692 high-quality proxy records from a wide range of natural paleoclimate archives at 648 locations. These are annual series of temperature-sensitive records that range from 50 to 2000 years back in time and are free to be used for temperature reconstructions of the CE.

The CESM-LME Project^19^ has released 36 last millennium simulations for 850–2005 CE from NCAR’s CESM-CAM5_CN general circulation model, 13 of them including all transient forcings (solar radiation, volcanic aerosols, greenhouse gases, land use/land cover conditions and orbital parameters). The remaining runs are ensembles of single-forcing simulations for the last millennium with only one transient forcing (either solar, volcanic, greenhouse gases, land use/land cover or ozone) and one control simulation (with forcings fixed at 1850 CE). Annual mean air temperature fields at 2 m (TREFHT) and 1.9° × 2.5° spatial resolution for the 850–2005 CE period of the full-forcing CESM-LME have been used as testbed to carry out the experiments with pseudo-proxies. For an arbitrarily chosen full-forcing member of the CESM-LME (the first one), we extracted the temperature series at the 569 grid points that include the locations of the PAGES-2k proxies, and used them as pseudo-proxies for the reconstruction of the target field (the 850–2005 CE global temperature fields of the same member). Clear-sky net solar flux at top of atmosphere (FSNTOAC, in Wm^−2^) from the control and single-forcing simulations have also been used to identify the dominant forcing for each year of the last millennium. In addition, SLP fields from the full-forcing simulations were employed to compute the Northern Annual Mode, defined as the first empirical orthogonal function of annual mean area-weighted SLP anomalies for [20–90] °N and 850–2005 CE.

To test the independence of the results with respect to the model employed, experiments have also been performed with 2 m air temperature fields from the CCC400, an ECHAM5.4 model ensemble^10,29^ composed of 30 fully forced members for the period 1601–2005 CE at 2° spatial resolution. Pseudo-proxies have been extracted from the first member of the ensemble at 569 grid points containing the locations of the PAGES-2k multi-proxy network.

Sensitivity tests have been carried out in real datasets and different climate realms. They include the 4.3°×5.7° proxy-based annual temperature reconstruction of the Last Millennium Reanalysis^9,38^ (LMR), based on the assimilation of paleoclimate records for 850–2000 CE from the PAGES-2k multi-proxy database and 2290 additional series from tree-rings, lake core, ice core, coral and speleothem archives. The LMR uses a prior state vector from CCSM4 model outputs, later modified by the assimilation of proxy records. We have also employed a complete global grid of annual temperature observations^30^ at 5° × 5° resolution for the industrial period (1850–2008 CE) by using an in-filled version of the HadCRUT4.2 database via the GraphEM algorithm^8^. From these gridded datasets we extracted the temperature series for the 569 CESM-LME pseudo-proxy locations. As the spatial resolution of the reconstructed and observed datasets is lower than that of the CESM-LME, some of these locations are situated over the same grid cell, reducing the effective number of grid points to 235 and 367, respectively.

### Pseudo-Proxies

Two types of pseudo-proxies have been constructed in the first full-forcing members of the CESM-LME and CCC400. They include perfect pseudo-proxies (simulated annual temperature series at the model grid points including the PAGES-2k locations) and more realistic pseudo-proxies^5,20,21^ with observational error, derived by adding red noise to the temperature series with a lag-1 Autoregressive Model (AR1). Pseudo-proxies have been synthetized for each time-step, t, from local temperatures series^5^. Different levels of SNR have been tested, including values such as infinite (perfect pseudo-proxies), 1 and 0.5. Note that pseudo-proxies are only sensitive to temperature and the observational availability is complete for the entire period.

### Climate field reconstruction methods

Global annual temperature fields for the 850–2005 CE period of the first CESM-LME full-forcing member have been reconstructed using the AM^20^, which provides spatially resolved CFRs from the limited sample of pseudo-proxy records. For each year, we sorted (from best to worst) analogues of the target simulation from a pool formed by the remaining 12 last millennium members of the full-forcing ensemble. Best analogues are the years of the pool with minimum RMSE for the pseudo-proxy locations. For each target year, the global temperature patterns of the best analogues are averaged at each grid point and taken as the reconstructed global field. The correlation between the reconstructed and target fields increases with the number of analogues employed but, at the same time, the variability ratio decreases (Fig. S16). Accordingly, we retained the three best analogues of each year, which is similar to those used in previous studies^20^. The sensitivity of the AM to proxy weighting was also tested by using area-weighted and un-weighted fields before the reconstruction procedure. Similar results were found, and hence no area weighting was applied, for coherence with^20^. The AM was also employed to derive NAM reconstructions for the first ensemble member from the temperature field over the pseudo-proxy locations. The reconstructed NAM is retrieved separately for each year by randomly picking the SLP field of one of the 100 best analogue years of the target temperature field of that year. This yielded 100 different SLP reconstructions for 850–2005 CE, with their corresponding NAM series.

On the other hand, the CCA has also been used in order to prove the independence of the results with respect to the reconstruction method. This method^25^ is based on maximizing the correlation (specifically the canonical correlation coefficients) of reduced eigenvectors from the pseudo-proxy and calibration datasets, the latter defined as the annual temperature field of the target simulation for the 1850–2005 CE interval. Results are similar if we use more realistic intervals for the calibration and validation periods (e.g. 1900–2005 CE and 1850–1899 CE, respectively). All temperature series are standardized by subtracting the mean and dividing by the standard deviation during the calibration period. To avoid degenerated eigenvalues (those with a value close to 0), both datasets have been truncated to a maximum number of 74 eigenvectors, which together explain more than 90% of the total variance. This corresponds to a minimum tolerance of 0.01 (eigenvalues below this threshold are discarded). Similar results are obtained for small changes in the dimensionality of the CCA. Note that the CCA usually generates reconstructions with smaller RMSE for the full 850–2005 CE period than the AM because temperature fields for the calibration period of the target simulation are employed in the reconstruction. Differently, the AM does not require calibration and therefore skill metrics can be computed for the entire time interval. The performance of the CCA during the validation interval was similar to the reconstruction skills retrieved for the calibration period and the remaining part of the last millennium (differences in area-weighted RMSE smaller than 0.1 °C).

### Evolutionary algorithm

Evolutionary algorithms are soft-computing techniques inspired by biological processes such as genetic recombinations and mutations that ensure the survival and evolution of best suited individuals within a natural competitive environment^39^. The CRO^22^ is a multi-method algorithm that combines different search strategies within a single population (a set of different solutions), where each substrate stands for a different search operator that recombines solutions in a different manner^40^ (e.g. 1-point crossover, multiple-point crossover, differential evolution, etc.). This, together with a small probability of selecting random locations (mutations), provides a wide range of new solutions sampled over the entire parameter space. In this case, we used the CRO to search for subsets of representative pseudo-proxy locations of the PAGES-2k network that optimize the RMSE between the target field and its reconstruction, obtained from different CFR methods. Corals (potential solutions to the problem) with smaller RMSE (known as the health function) will have more probability of surviving for successive iterations (generations), promoting the recombination of best sets of locations in the long-term. In our case, good enough solutions converged after 500 generations. Unlike sequential approaches (methods employed to solve linear problems by adding successively individual elements), the CRO looks for efficient solutions simultaneously (i.e. it selects the set of locations “all-at-once”), which makes it highly parallelizable for the exploration and optimization of non-linear systems. As in many other combinatorial problems, the method does not guarantee the best possible solution (assuming that it is unique) but an optimized one, and hence slightly different solutions can be found for different runs of the algorithm. In our experiments, these solutions, although different, keep the same spatial structure, indicating that the selected regions are indeed meaningful.

### External forcings

To determine if a particular forcing is dominant for a certain year, we have used the area-weighted mean FSNTOAC from the control simulation and the ensemble of single-forcing simulations for the following available forcings: orbital, solar, volcanic, land use/land cover, greenhouse gas concentrations and ozone. For each year and single-forcing run, we computed the absolute FSNTOAC difference with respect to the 1156-yr mean of the control simulation. By doing this, we estimated the ensemble mean radiative imbalance for each year and forcing. A given year is assigned to an external forcing if two conditions are met: that the forcing has the highest absolute FSNTOAC difference, and this value is above 2 standard deviations of the control mean (to avoid false forcing assignations), which provides a measure of internal variability. Otherwise, the year is not assigned to any external forcing, this subset comprising years of weak or multiple external forcings, which may obscure the attribution exercise. With this approach, ~60% of years on record could not be assigned to a single forcing, whereas ~21% and ~10% of years were associated with volcanic and solar forcings, respectively. On the other hand, only ~2% of years on record have been associated with greenhouse gas emissions (note that this forcing is not relevant before 1850 CE). The remaining forcings, namely land use/land cover (~4%), ozone (~2%) and orbital (less than 1%), dominated for very few years and are not considered, since their frequency series for the last millennium did not show signals discernible from the internal variability. Note that this attribution method is instantaneous (i.e. it assigns dominant forcings year by year), and not fully independent since forcing years are selected from different simulations of the same model.

### Statistical significance

False detection tests^41^ regarding serial correlation and test multiplicity have been applied for the determination of global statistical significance in correlation maps between the reconstructed and target fields (Fig. 2 of the main text, and Fig. S9). Because of the large N, effective degrees of freedom are high, leading to significant correlations at the 95% confidence level for all grid points with significant differences in Figs. 2 and S9. Furthermore, False Discovery Rates (FDRs) have been calculated to assess whether there are falsely attributed significant correlations^41^. In all cases, correlation maps passed the multiplicity test for a FDR of 5%. Note that these tests have been performed for pseudo-proxy experiments under idealized conditions, and the high statistical significance of the results is not necessarily representative of real proxy-based reconstructions, where additional sources of uncertainty are present.

## Supplementary information

Supplementary information.

## Data Availability

All datasets used for this study are publicly available. Climate variables of the CESM-LME full-forcing ensemble can be found CESM projects website (www.cesm.ucar.edu/projects/community-projects/LME), whereas the PAGES-2k Network Temperature Database can be downloaded from Figshare (https://doi.org/10.6084/m9.figshare.c.3285353.v1). HadCRUT temperature observations are included in the PAGES-2k database as the calibration datasets, and Last Millennium Reanalysis outputs can be found the LMR Project Data Page (https://atmos.washington.edu/~hakim/LMR).

## Code availability

The code for the evolutionary algorithm is available upon request.
