# Supplementary material for: Selection of optimal proxy locations for temperature field reconstructions using evolutionary algorithms
Source: Sci Rep. 2020 May 13;10:7900. doi: 10.1038/s41598-020-64459-6 (PMC7221084; doi:10.1038/s41598-020-64459-6)
Supplement: Supplementary file 1 — Supplementary information. [file 41598_2020_64459_MOESM1_ESM.pdf]

SUPPLEMENTARY INFORMATION

Selection of optimal proxy locations for temperature field reconstructions using evolutionary algorithms

*Fernando Jaume-Santero[1,2], David Barriopedro[2], Ricardo García-Herrera[1,2], Natalia Calvo[1], Sancho Salcedo-Sanz[3]

[1]*Department of Earth Physics and Astrophysics, Universidad Complutense de Madrid, Madrid, Spain*

[2]*Geosciences Institute (IGEO), (CSIC/UCM), Madrid, Spain*

[3]*Department of Signal Processing and Communications, Universidad de Alcalá, Madrid, Spain*

Supplementary Tables

Acronym	Description
CRO	Coral Reef Optimization algorithm
CRO-AM	Coral Reef Optimization coupled with the Analogue Method
CRO-CCA	Coral Reef Optimization coupled with the Canonical Correlation Analysis
CRO-MIN	Minimum subset of perfect pseudo-proxies obtained with CRO-AM necessary to outperform the reconstruction skill of the full-proxy network
CRO-OPT	Optimized subset of perfect pseudo-proxies of the PAGES-2k network obtained with CRO-AM that yields the best reconstruction skill

Supplementary Table S1 | List of acronyms with the CRO algorithm included in the text.

	MCA-LIA Temperature (ºC)	
Map	Global	NH
Target	0.19	0.20
CRO-MIN (17)	0.08	0.10
CRO-OPT (120)	0.11	0.13
PAGES-2k (569)	0.09	0.11

Supplementary Table S2 | GMT differences between the Medieval Warm Period (950-1250 CE) and the Little Ice Age (1500-1850 CE). Area-weighted mean temperatures are calculated globally and for the Northern Hemisphere (NH). The target is the first ensemble member of the CESM-LME. Reconstructions are generated with CRO-AM using perfect pseudo-proxies at the locations of CRO-MIN, CRO-OPT and the full-proxy network of PAGES-2k.

Supplementary Figures

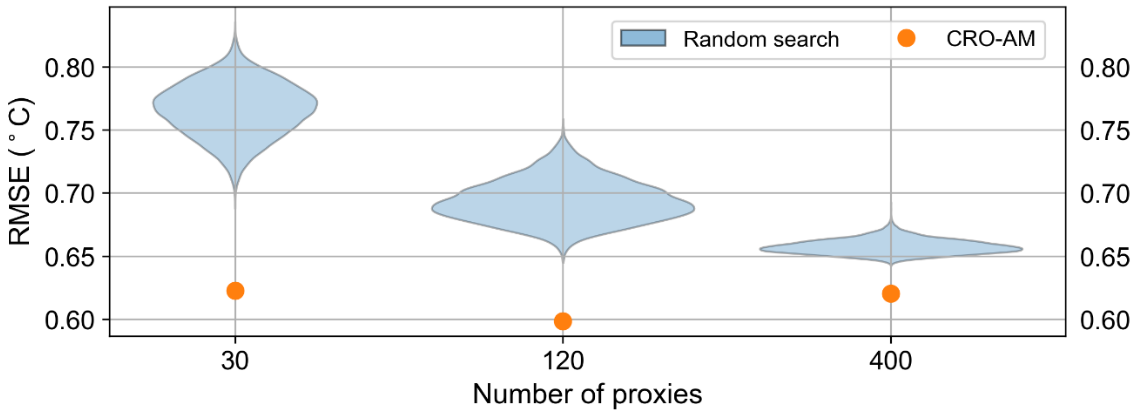

Supplementary Figure S1 | RMSE of 850-2005 CE global temperature fields reconstructed with different subsets of perfect pseudo-proxies from the PAGES-2k network. Orange dots represent the RMSE associated with the reconstructions using the optimized subsets of 30, 120, and 400 perfect pseudo-proxies of the PAGES-2k network obtained with the CRO-AM. Blue violins show the RMSE distribution obtained from 10000 reconstructions using different combinations of 30, 120, and 400 randomly selected pseudo-proxies from the PAGES-2k network. RMSE are calculated with respect to the global temperature fields of the target simulation (the first member of the CESM-LME).

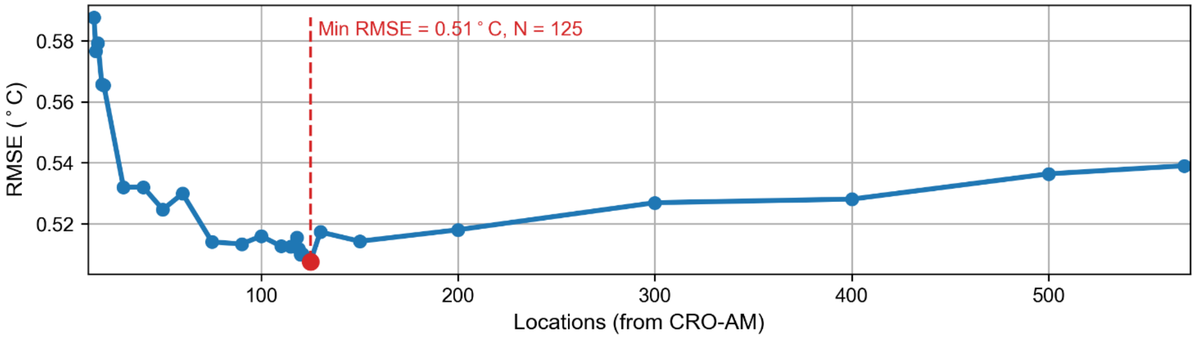

Supplementary Figure S2 | RMSE of CCA reconstructions generated with the optimized subsets of perfect pseudo-proxies of the PAGES-2k network selected by the CRO-AM. Red dot and dashed line highlight the minimum RMSE. Compare this figure with Fig. 1 of the main text.

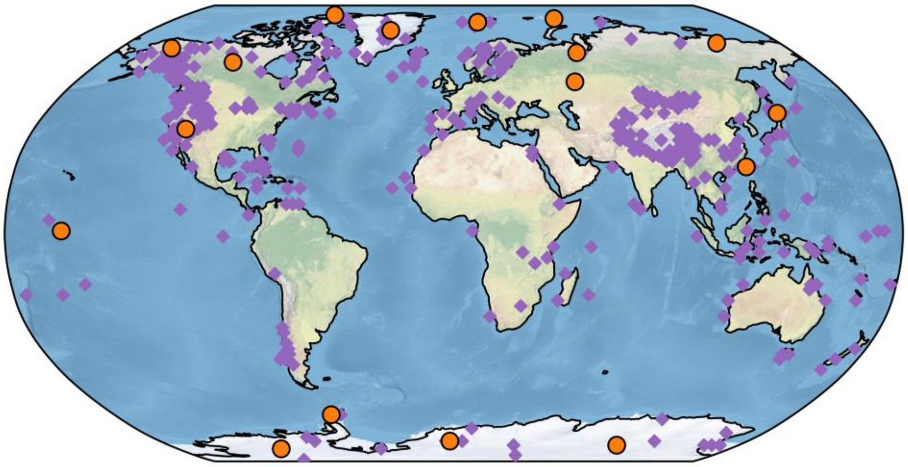

Supplementary Figure S3 | Minimum subset of perfect pseudo-proxies (CRO-MIN, orange dots) needed to obtain a CRO-AM reconstruction with lower RMSE than that of the full-proxy PAGES-2k network (purple diamonds).

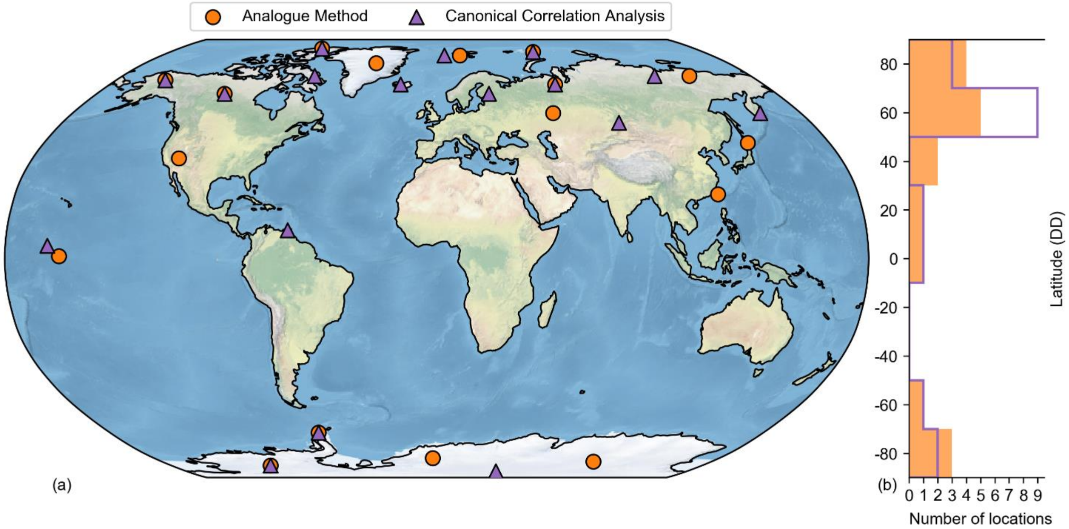

Supplementary Figure S4 | Optimized subsets of 17 perfect pseudo-proxies of the PAGES-2k network selected by CRO-AM (CRO-MIN) and CRO-CCA. a, 2-D and **b,** latitudinal distributions of the CRO-MIN locations obtained with CRO-AM (orange dots and shading) and the corresponding subset of perfect pseudo-proxies of the PAGES-2k network (with the same size as CRO-MIN) obtained with CRO-CCA (purple diamonds and line).

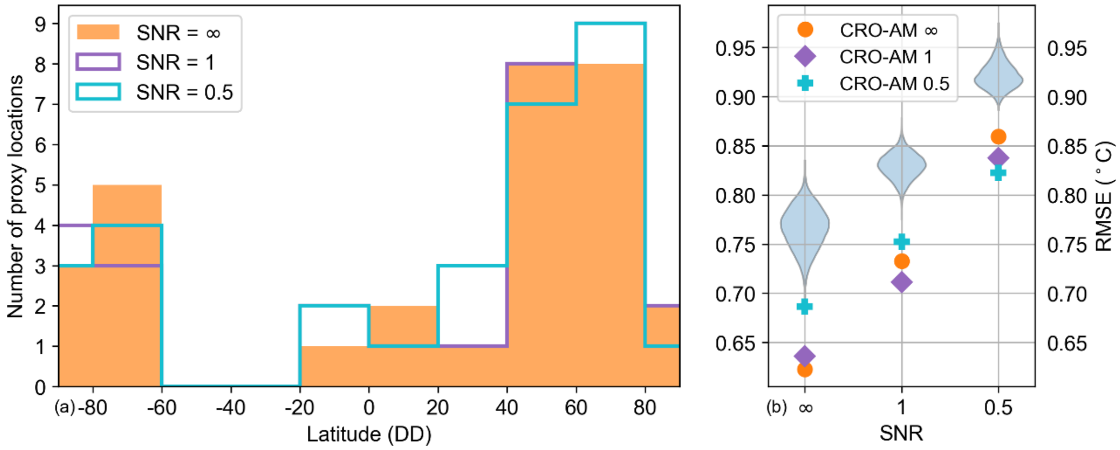

Supplementary Figure S5 | Sensitivity of CRO-AM reconstructions to pseudo-proxies with different levels of observational error. a, Latitudinal distribution of the optimized subsets of 30 locations selected by CRO-AM from a PAGES-2k network of perfect pseudo-proxies (SNR = ∞, orange shading), and noisy pseudo-proxies with SNR = 1 (purple line) and SNR = 0.5 (blue line). **b,** RMSE of CRO-AM reconstructions from pseudo-proxies with different SNR. For each type of pseudo-proxies, symbols indicate the RMSE of the reconstruction obtained with the optimized subsets of locations found for perfect pseudo-proxies (orange dots), and noisy pseudo-proxies with SNR of 1 (purple diamonds) and 0.5 (blue crosses). Blue violins illustrate the RMSE distributions of 10000 reconstructions obtained from subsets of 30 PAGES-2k locations selected at random.

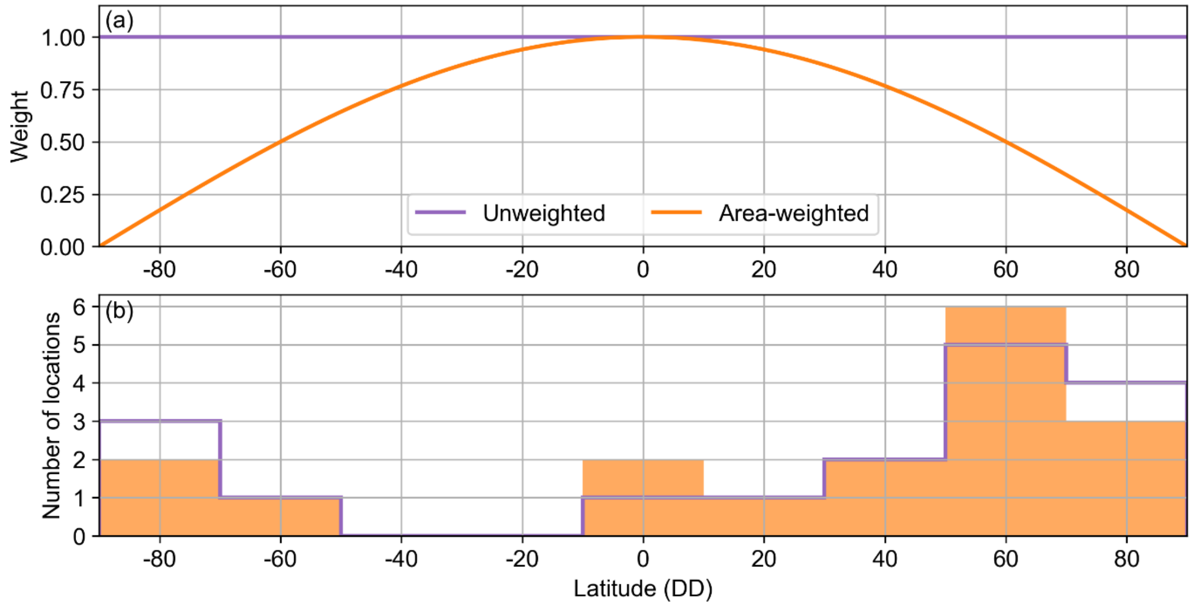

Supplementary Figure S6 | Sensitivity of the optimization process to area-weighting. a, Weights assigned to perfect pseudo-proxies as function of their latitude in two experiments of the CRO-AM. **b,** Latitudinal distribution of optimized subsets of 17 perfect pseudo-proxies from the PAGES-2k network obtained with area-weighted (orange shading) and unweighted (purple line, CRO-MIN) versions of CRO-AM.

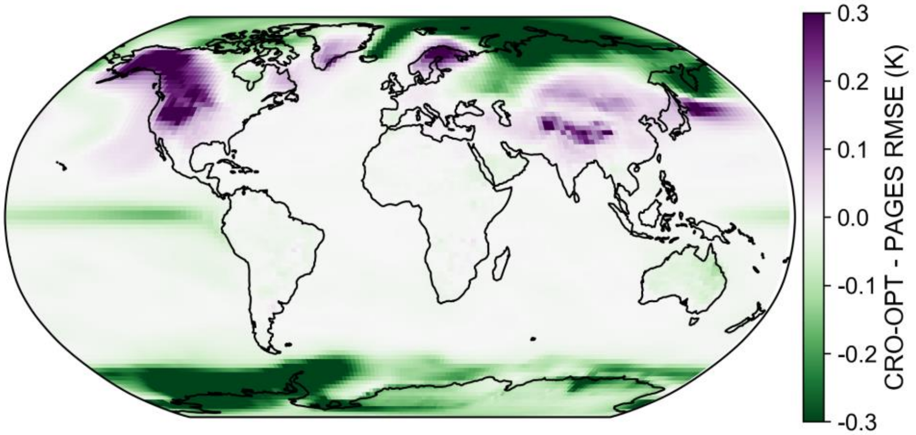

Supplementary Figure S7 | RMSE difference between the CRO-OPT and full-proxy reconstructions. Green (purple) color illustrates regions where CRO-OPT yields lower (higher) RMSE than the reconstruction with the full PAGES-2k network of perfect pseudo-proxies. RMSE are calculated with respect to the target field (the first CESM-LME member). Note that RMSE is not fully independent from the Pearson correlation coefficient displayed in Fig. 2b of the main text.

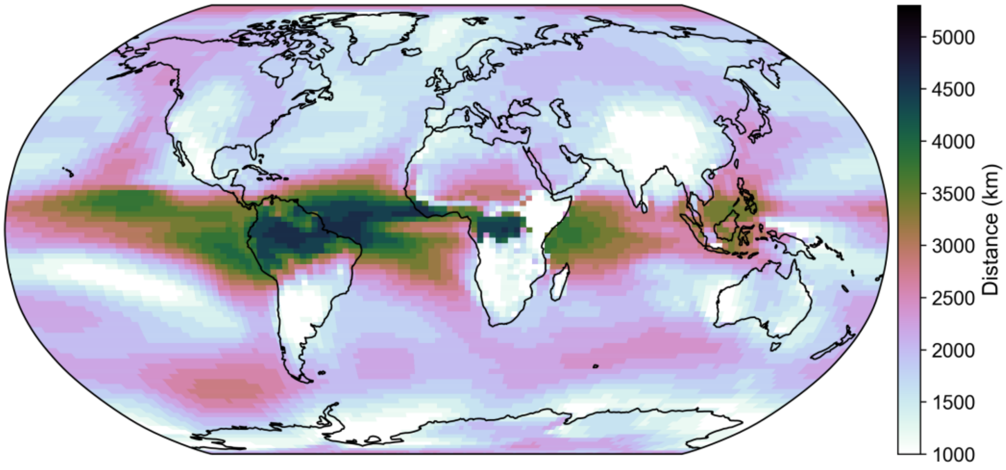

Supplementary Figure S8 | Spatial map of e-folding distances of decorrelation for the annual temperature of the first full-forcing CESM-LME member. The distance (in kilometers) for each grid point defines the area of the circle for which the averaged coefficient of determination (R^2) has decayed below e^{-1}.

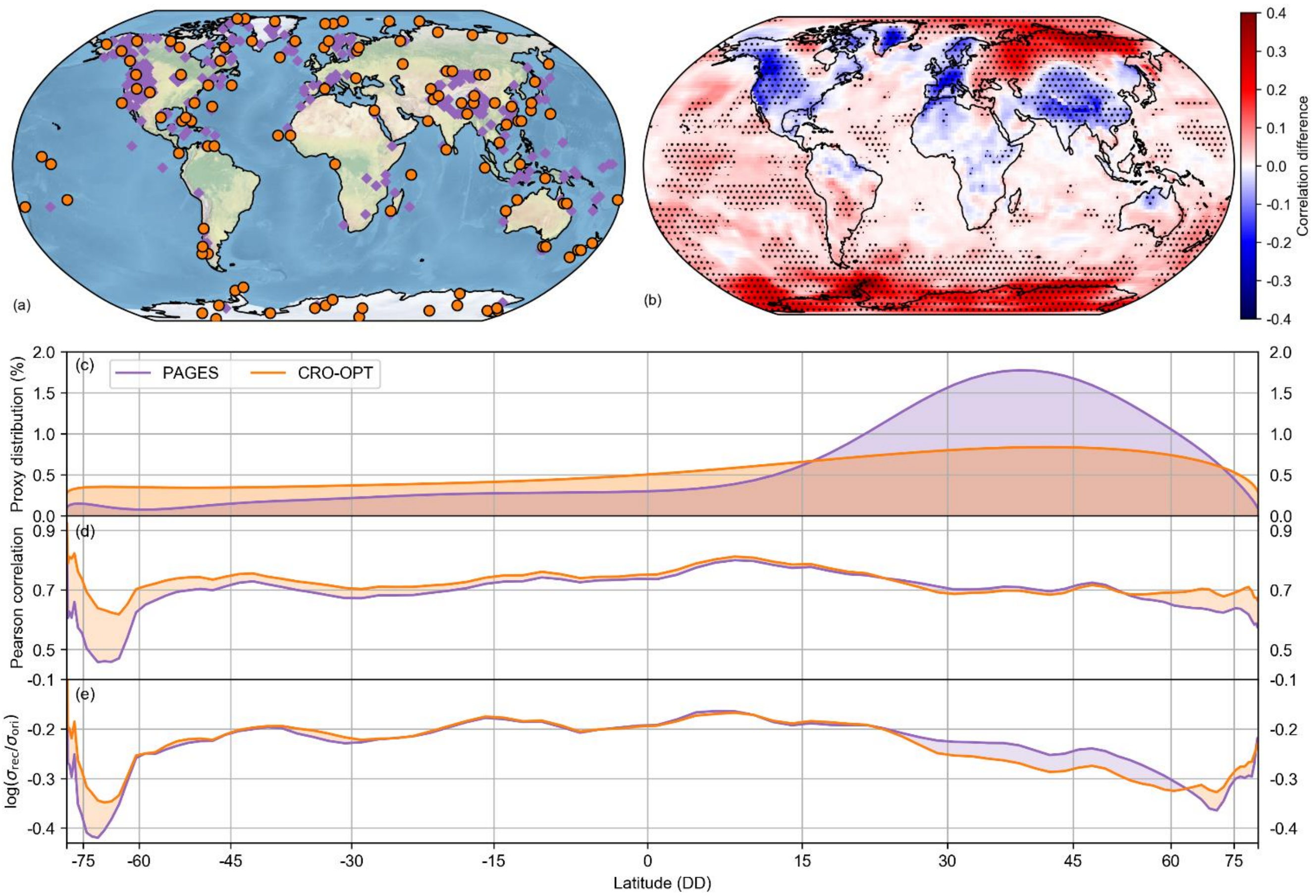

Supplementary Figure S9 | As Figure 2 of the main text but using the global temperature fields of the CCC400 first ensemble member (1601-2005 CE) as target. The reconstruction has been obtained from the optimized subset of perfect pseudo-proxies of the PAGES-2k network (with the same size as CRO-OPT) selected by CRO-AM in the CCC400 model ensemble.

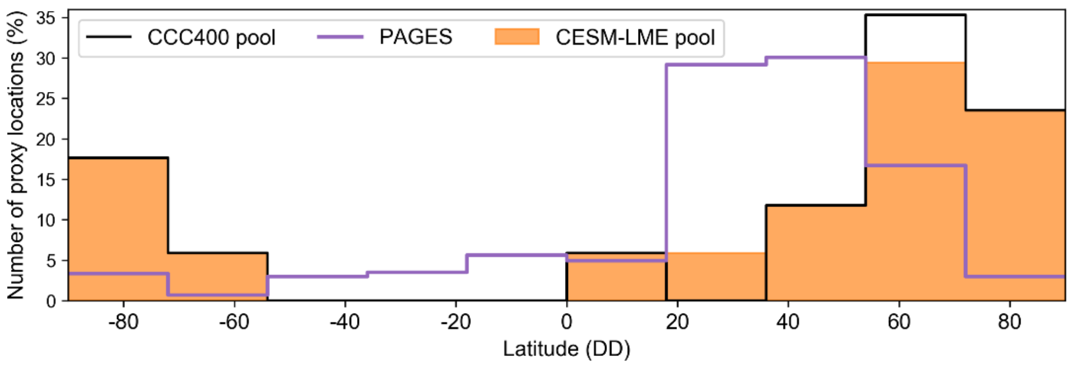

Supplementary Figure S10 | Latitudinal distribution of optimized subsets of the PAGES-2k network using different model ensembles as a pool for the CRO-AM reconstruction of the first CESM-LME member. Each subset includes 17 perfect pseudo-proxies obtained with the CRO-AM using as a pool members of the CESM-LME (orange shading) and the CCC400 ensemble (black line). In both cases, the target is the 850-2005 CE global temperature fields of the first member of the CESM-LME. The purple line illustrates the distribution of full-proxy PAGES-2k network.

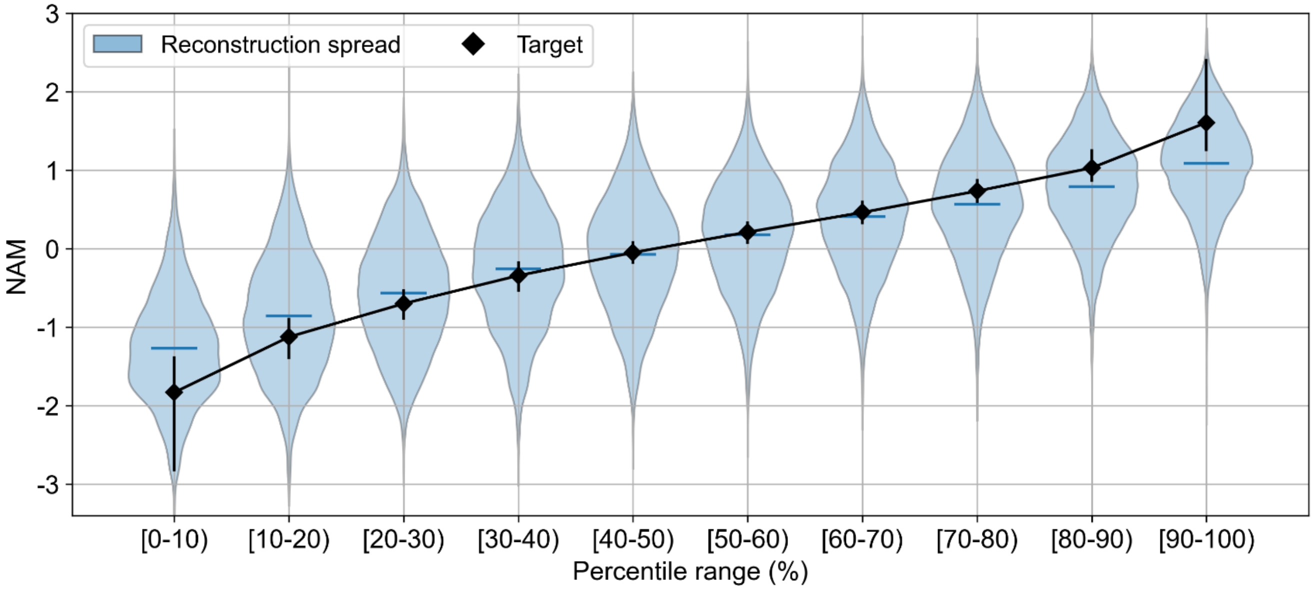

Supplementary Figure S11 | Percentile distribution of simulated NAM values in the first CESM-LME member (850-2005 CE) and their corresponding reconstructions from the CRO-OPT subset of the PAGES-2k network. Black diamonds represent the mean simulated NAM for each percentile range, with vertical black lines showing their respective minimum and maximum values. Blue violins show the distribution of the reconstructed NAM values for the same years included in each percentile range and 100 different NAM reconstructions. Mean values of the violin distributions are depicted as horizontal blue lines. See Methods for details.

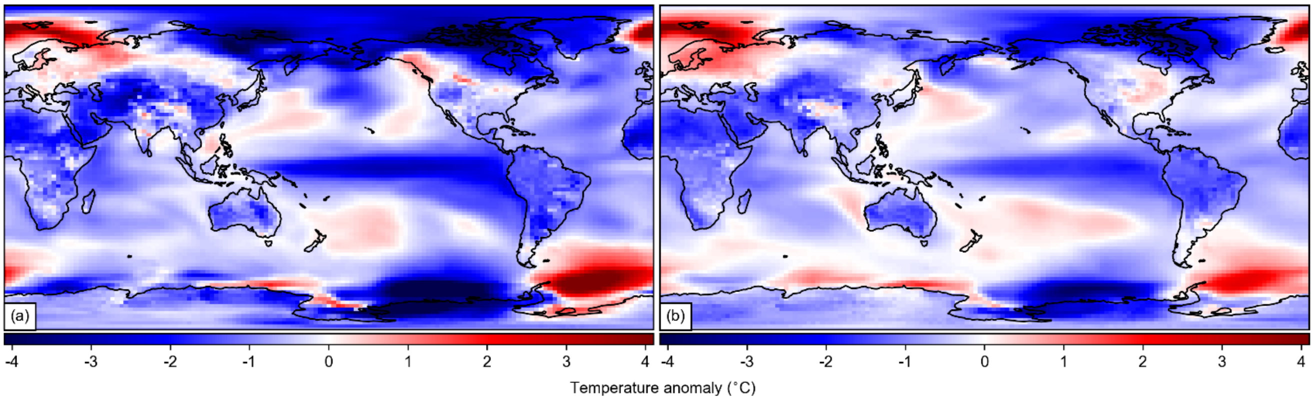

Supplementary Figure S12 | Annual temperature anomalies (ºC) after Tambora's eruption (1815 CE) in a, target simulation and b, CRO-OPT reconstruction. Anomalies are calculated as the difference between the year after the eruption and the mean temperature of the 2 previous years.

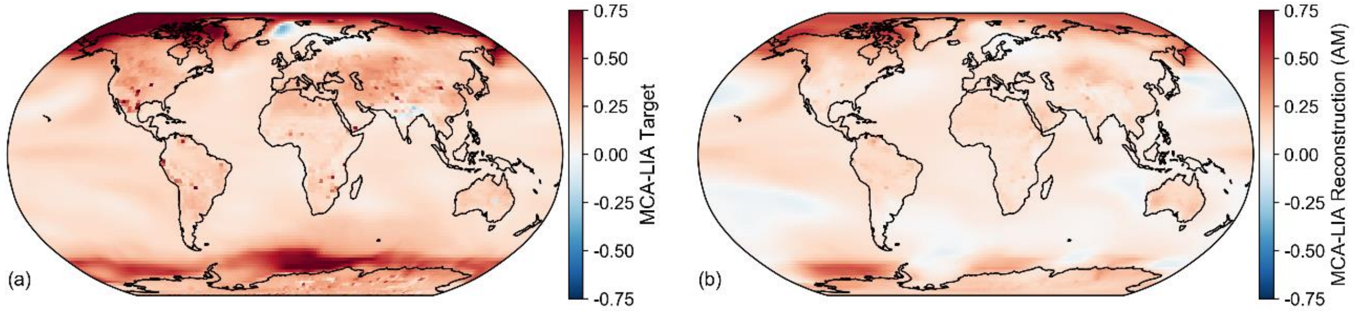

Supplementary Figure S13 | Spatial pattern of mean temperature difference (ºC) between the MCA (950-1250 CE) and LIA (1450-1850 CE) in a, the target simulation and b, CRO-OPT reconstruction.

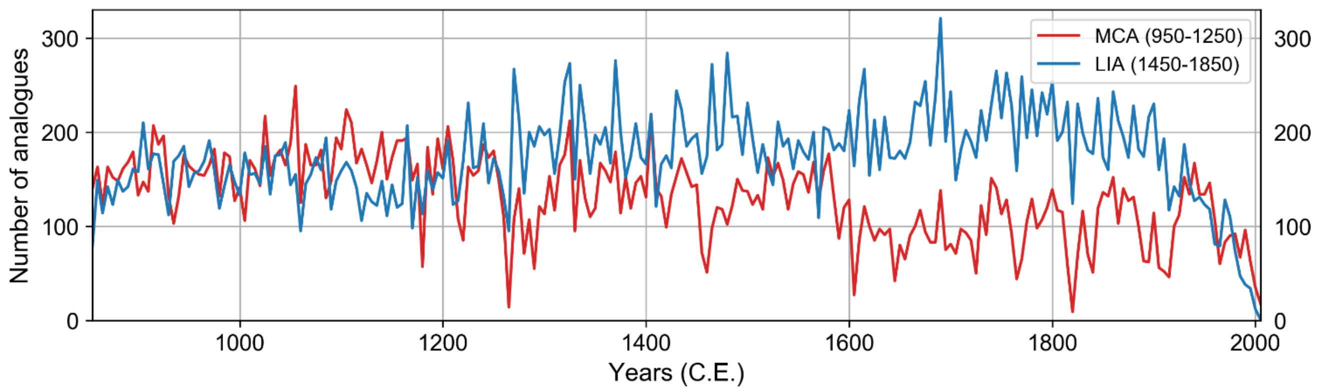

Supplementary Figure S14 | Time series with the total frequency of analogues for all years of the MCA (950-1250 CE, red) and LIA (1450-1850 CE, blue) in the CRO-OPT reconstruction. For each year of the MCA and LIA in the target simulation, the 100 best analogues of the annual temperature at the CRO-OPT locations are selected. Their respective years of occurrence are retained and accumulated through the MCA and LIA periods, separately.

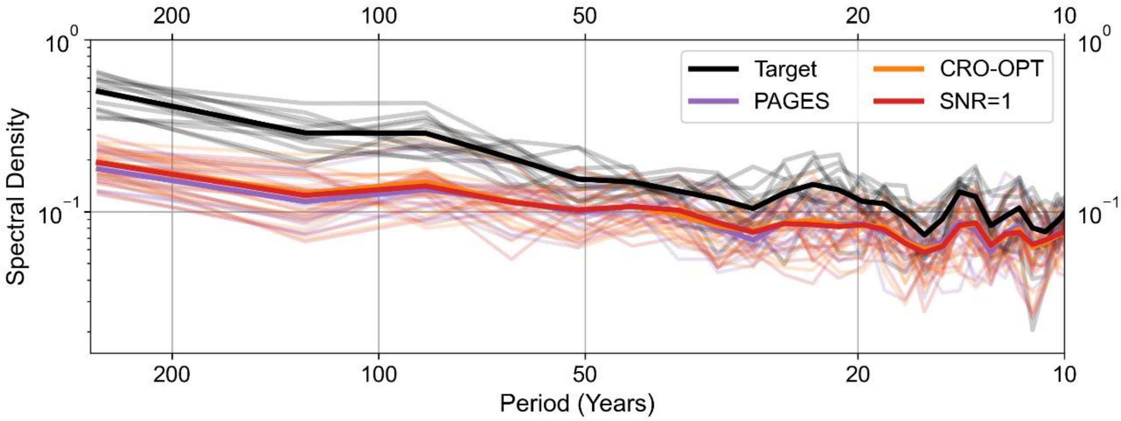

Supplementary Figure S15 | Power density spectrum of GMTs series for 850-2005 CE. Color lines show the power spectrum of area-weighted global mean temperature anomalies calculated for the target simulation (black), the CRO-OPT reconstruction (orange), and the CRO-AM reconstructions generated with the full-proxy PAGES-2k network of perfect pseudo-proxies (purple), and an optimized subset of 150 noisy pseudo-proxies with SNR=1 (red). Opaque lines depict the mean spectral density of the ensemble, and transparent lines represent individual spectral densities of all (13) members of the ensemble

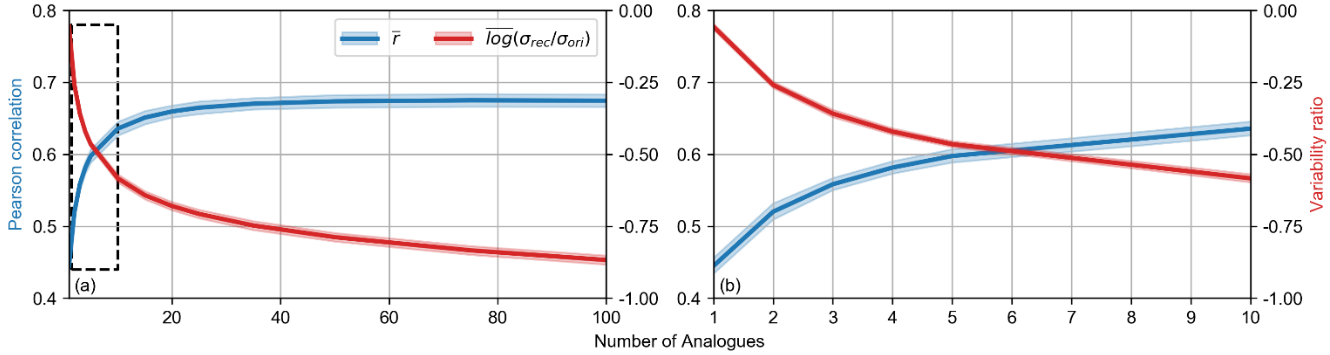

Supplementary Figure S16 | Pearson correlation (blue) and variability ratio (red) for AM reconstructions of global temperature fields as a function of the number of analogues. For each member of the CESM-LME the remaining 12 full-forcing simulations are used to reconstruct the global temperature fields from the full-proxy PAGES-2k network of perfect pseudo-proxies, using different number of analogues. Shading shows the spread (two standard deviations with respect to the mean values). **a,** Correlation and variability ratio for AM reconstructions with 1 to 100 analogues. **b,** A zoom of the black dashed square in **a**.
